# Resolving severe oligohydramnios as an early prenatal presentation of renal coloboma syndrome—A report of two generations

**DOI:** 10.1002/ccr3.4758

**Published:** 2021-09-07

**Authors:** Andrew Nguyen, Carla Campagnolo, Ghislain Hardy, Maha Saleh

**Affiliations:** ^1^ Schulich School of Medicine and Dentistry University of Western Ontario London ON Canada; ^2^ Department of Paediatrics Division of Genetics and Metabolism London Health Sciences Centre London ON Canada; ^3^ Department of Obstetrics and Gynecology London Health Sciences Centre London ON Canada

**Keywords:** diagnosis, genetic testing, oligohydramnios, PAX2, prenatal presentation, renal coloboma syndrome

## Abstract

This report suggests that self‐resolving oligohydramnios is an early sign of malfunctioning kidney in individuals with renal coloboma syndrome (RCS) and demonstrates how a genetic diagnosis can impact patient and fetal management as it outlines two generations of RCS.

## Graphical abstract

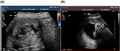

## 1 INTRODUCTION

Renal coloboma syndrome (RCS) is an autosomal dominant disorder with high penetrance and variable expressivity.^1^ RCS is primarily characterized by renal dysplasia and abnormalities of the optic nerve.^1,2^

Although there are no formal diagnostic criteria for RCS, it is thought to be associated with a heterozygous pathogenic variant in *PAX2*.^1^ 50% of patients with clinical findings suggestive of RCS (eg, renal and ocular malformations) have a variant in the *PAX2* gene.^2^ As a result of improved access to molecular genetic testing, more individuals with *PAX2* variants have been identified with a wide phenotypic variability.

Renal and ocular findings are reported in 92% and 72% of affected individuals, respectively.^1^ Abnormalities in kidney structure and function are among the most frequent findings in individuals with *PAX2* variants, which include renal dysplasia, multicystic dysplastic kidney, and end‐stage renal disease. Primary ocular findings involve optic nerve dysplasia that ranges from severe to mild.^1^, ^2^

Reports of prenatal RCS findings have been limited, highlighting the importance of intrafamilial monitoring among affected individuals. Once a *PAX2* pathogenic variant has been identified in a family, prenatal genetic testing for the familial variant becomes possible, in addition to screening through ultrasound (US), to detect any renal malformations and assess amniotic fluid levels.^1^ To date, only six case reports and one large series have been published on prenatal RCS findings.^3^, ^4^, ^5^, ^6^, ^7^, ^8^ Of the six case reports, four involved terminated pregnancies as a result of prenatal US findings of fetal renal dysplasia and/or oligohydramnios.^3^, ^4^, ^5^ Common findings among all case reports include bilateral fetal renal hypoplasia and observed bilateral optic disc colobomas postnatally. Upon genetic testing, there is considerable variability in the *PAX2* gene variants, with most case reports having a de novo variant.^4^, ^5^, ^7^, ^8^ Notably, one case report presents a male with moderate‐severe oligohydramnios for which an amnio‐infusion of 250 ml of normal saline was performed. The remainder of the pregnancy was monitored with serial US examinations, leading to a live birth following that intervention.^3^ Bower et al reported one of the biggest RCS prenatal case series that added 13 prenatal cases to the literature.^9^ Six of the 13 fetuses were terminated given prenatal findings of renal hypoplasia, in addition to severe oligohydramnios. All six cases had parents confirmed with a pathogenic *PAX2* variant. The other seven surviving cases had de novo variants. Five out of the seven had normal amniotic fluid levels, while the other two had mild oligohydramnios, in addition to a renal hypoplasia or dysplasia. None of the 13 cases reviewed had self‐resolving oligohydramnios.

We further add to the literature a report of mother and her newborn with RCS, both presenting prenatally with self‐resolving severe oligohydramnios as an early prenatal finding.

## 2 CASE

**FIGURE 1:**
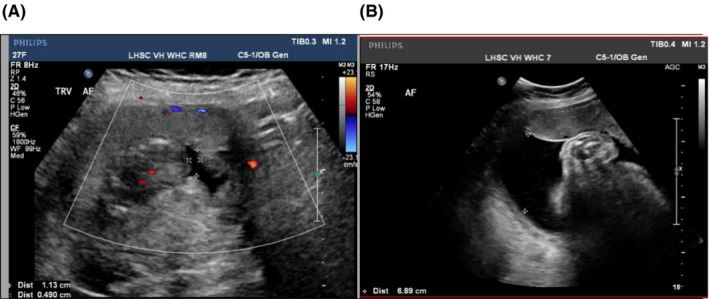
A, Antenatal ultrasound image at 16 weeks and 1 day gestation showing a transverse view of the uterus with severe oligohydramnios. B, Antenatal ultrasound image at 36 weeks gestation showing normal amniotic fluid with a maximum vertical pocket of 6.89 cm

The proband was a 27‐year‐old primigravida who was referred to the Maternal Fetal Medicine (MFM) clinic given a personal history of obesity and chronic renal disease post‐transplant. She was seen at 16 weeks and 1 day gestation with findings of severe oligohydramnios on a viability US. (Figure 1A). A limited survey of the anatomy was attempted during which the bladder and fetal kidneys were not well visualized due to the low amniotic fluid, large maternal body habitus, and the early gestational age. The patient denied any symptoms of vaginal fluid loss and had no known exposure to teratogens or any relevant medications (eg, NSAIDs or ACE inhibitors).

The patient's medical history was remarkable as she was personally induced near term, given early prenatal history of anhydramnios that “normalized” as reported by her mother. She was subsequently diagnosed with a solitary dysplastic left kidney. She had a right ureteropelvic junction (UPJ) obstruction and a progressive segmental sclerosis, leading to end‐stage renal disease at 20 years of age. As such, she required a kidney transplant the year prior to this pregnancy.

A three‐generation pedigree was reviewed. Our patient was the second child born to non‐consanguineous couple of Irish and French‐Canadian descent. Her older sibling had 3 healthy children and terminated a pregnancy with an isolated hypoplastic left heart. Her partner is one of three healthy siblings of English and Dutch background.

A follow‐up with the multi‐disciplinary clinic (MFM, NICU, and Genetics) was planned with a repeat US performed at 18+5 weeks gestation, again showing severe oligohydramnios with a maximum vertical pocket of 1.1 cm. The bladder was visualized, but the kidneys and renal arteries were not clearly seen. There were no anomalies identified in that US, and growth was found to be adequate for the given gestational age. The couple was counseled by the team on the likely poor perinatal prognosis given the severe oligohydramnios in the mid‐trimester and its association with pulmonary hypoplasia. Prenatal investigation options were discussed, and a pregnancy termination option was presented with early induction, postnatal autopsy, and testing. The couple opted for expected management and was well aware of the high likelihood of poor perinatal outcomes and the need for close follow‐up.

The fetal kidney and renal artery were seen in a repeat US at 21 weeks gestation and appeared grossly normal despite being on the smaller size. Subsequent biweekly US showed gradual improvement of fluid levels.

Given her medical history and constellation of features, the patient was offered a Congenital Abnormalities of the Kidney and Urinary Tract (CAKUT) next‐generation sequencing panel, which revealed a pathogenic variant involving the *PAX2* gene described as c.76dup: p. Val26Glyfs*28: NM_003990.4. This is responsible for the clinical condition of RCS, also described as a *PAX2*‐related disorder.

The timing of the results coincided with a repeat US at 36 weeks in which amniotic fluid levels normalized (Figure 1B). Around that time, our patient started experiencing abnormal vision in the context of a headache. Recognizing the ocular spectrum of RCS, this prompted an urgent ocular assessment. Dilated fundus examination of both eyes revealed no retinal tears or detachments. No ocular coloboma was observed; however, there was vitreous syneresis and dilated vessels in the right eye. Ocular follow‐up was recommended, given the RCS diagnosis and risk for future eye concerns. Knowing RCS’s autosomal dominant inheritance, that diagnosis was suspected for the baby. Postnatal kidney US and eye examination were planned for the newborn, in addition to targeted testing for the *PAX2* variant.

The amniotic fluid levels had normalized in the beginning of the third trimester and remained in a similar range up to the time of delivery. The patient was induced at 37 weeks and 2 days gestation and had a baby girl weighing 7 pounds 11 ounces via an emergent cesarean section. The baby's eye examination was normal. Her bloodwork showed slightly elevated serum creatinine, partially reflecting maternal serum creatinine, age‐appropriate estimated glomerular filtration rate, some albuminuria, and no concern about hypertension or pulmonary hypoplasia. Her kidneys were normal in morphology on US, with a lower end of measurement for her age. Genetic testing confirmed the maternal *PAX2* variant. Upon a recent check‐up, the almost 2‐year‐old girl had stable mild albuminuria with echogenic kidneys based on US, with suspected bilateral renal dysplasia, and continues to be monitored by nephrology.

## 3 DISCUSSION / CONCLUSION

Our case provides further evidence that oligohydramnios is an early US sign of malfunctioning kidneys in a fetus affected with RCS. Both the mother and her child were affected, and we saw that the low levels of amniotic fluid throughout the second trimester indicated kidney malfunction, despite renal structure initially appearing grossly normal as seen in the baby girl. For both patients, the amniotic fluid volume normalized in the early third trimester without intervention. Given the maternal personal history, the couple elected to continue their pregnancy as they were confident that the fluid levels would normalize and pulmonary insufficiency seemed unlikely. With this decision, we were able to document a two‐generation history of self‐resolving oligohydramnios as an early US sign of RCS.

We described earlier how RCS is known to be associated with a heterozygous pathogenic PAX2 variant. During embryonic development, the PAX2 gene is involved with the formation of many organs, including the developing kidneys. Debhi et al described the PAX2 gene's role during the mesenchymal‐to‐epithelial transition in renal development.^10^ To date, most cases flagged prenatally were identified due to renal hypoplasia secondary to PAX2 mutations.

Renal insufficiency and end‐stage renal disease (ESRD) without hypoplasia have been described by Bower et al, with the mean age at ESRD diagnosis in PAX2‐related conditions being 19.5 years.^1^ This age at diagnosis ranged from birth to 79 years. We propose that in our patient, the oligohydramnios was an early sign of renal malfunction. In the absence of renal hypoplasia, progressive increase in kidney size compensates for the malfunctioning podocytes, which resulted in a transient oligohydramnios.

This case also highlights how an RCS diagnosis through the CAKUT sequencing panel has impacted management for the mother who required an eye examination prior to her delivery. This molecular diagnosis prompted the nephrology team to closely monitor the newborn girl. Through this monitoring, we were able to see early signs of renal failure in the baby girl, with suspected bilateral kidney dysplasia on postnatal US, not noted until recently at almost 2 years of age.

Given the mother's RCS diagnosis and past medical, obstetrical, and surgical history, it puts her at high risk for obstetrical care. Shortly after giving birth, the mother returned to the high‐risk obstetrical clinic with her second pregnancy. Amniotic fluid levels were always normal in that subsequent pregnancy. Reassured, the couple deferred molecular testing for the *PAX2* variant until after delivery. The mother was able to successfully, without complication, deliver a second daughter who was not affected by the *PAX2* variant.

In this case, the collection of a detailed medical and family history, and subsequent genetic analysis related to the patient's history, led to a better understanding of the US anomalies seen during pregnancy and greatly impacted neonatal care.

**Table:** 

What is Already Known About This Topic?	What Does This Study Add?
There are no publications linking prenatally detected severe oligohydramnios that self‐corrects upon birth in RCS patients.	We report two generations of RCS, flagged prenatally, with resolving severe oligohydramnios. This report suggests that self‐resolving oligohydramnios is an early sign of malfunctioning kidney in RCS individuals and demonstrates how a genetic diagnosis impacted patient management.

## CONFLICTS OF INTEREST

We have no conflict of interests to disclose.

## AUTHOR CONTRIBUTIONS

Andrew Nguyen: Literature review, conceptualization, patient case review, manuscript write‐up, review and editing. Carla Campagnolo: Genetic counseling consultation, manuscript review and editing. Ghislain Hardy: OB/Gyn consultation, figures and captions, manuscript review and editing. Maha Saleh: Literature review, patient consultation, conceptualization, manuscript write‐up, review and editing. All authors discussed the results and contributed to the final manuscript.

## Data Availability

The data that support the findings of this study are available on request from the corresponding author. The data are not publicly available due to privacy or ethical restrictions.
